# Fathers Ancient and Modern

**Published:** 1891-06-13

**Authors:** 


### The Doctor's Armchair.

### FATHERS ANCIENT AND MODERN.

Mr. Herbert Spencer, in giving a certain measure of approval to the operations of the Society for the Prevention of Cruelty to Children, nevertheless ventures to hint a caution, lest one of the results of the Society's work should be the diminution of paternal responsibility. Mr. Spencer, with true insight, perceives that the father is the unit of power and guidance in the family; and is the natural complement of general State authority. It has never been more necessary than it is now that all real and acknowledged power in State and social affairs should be recognised, and placed, so to speak, on its proper eminence, so that all persons may know where authority is, and who is justly entitled to wield it. Such separation and conspicuousness of authority is necessary even in small states, and where the numbers of the population are very limited. But it is ten thousand times more necessary when populations are very large, and tend to increase with great rapidity. In such circumstances vast numbers of the people soon get almost entirely out of hand. They herd together in thickly crowded slums, where neither law, as represented by the magistrate and the policeman, nor religion as represented by the clergyman, nor public opinion as represented by the culture, morals, and the prevailing sentiments of the upper and middle classes, can have any practical influence upon them at all.

Masses of people thus circumstanced, can hardly fail to be degraded, To begin with, such masses consist of the least intelligent, the least enterprising, and the least moral of their kind. Most of the ordinary social influences which act upon small communities are practically non-existent for them. It, therefore, becomes all the more essential that all those natural forces of authority which cannot be separated from communities of people by any artificial means, should be sought out, strengthened, and encouraged to bear the utmost strain that can be put upon them.

It is obvious that wherever there is a community of persons, whatever be the degree of their civilization, or the state of their morals, there must be fathers. And it is equally obvious that fathers, by virtue of their superior reasoning powers and stronger personality, will, to a large extent and in a very practical way, bear rule in their households and in a certain degree outside them. We know, as a matter of fact, that among all ranks of society, and at almost every period of the world's history, fathers have been the ruling authorities in the small state which is included within the limits of the home. We know, moreover—at least, those of us know who are familiar with manufacturing towns and with the industrial portions of the metropolis—that paternal authority tends to decrease in the direct ratio of the increase of the population. The more rapidly a people increases, the earlier are the children put to work; and just as rapidly as the power of self support develops in a child, so rapidly does the regard for paternal authority diminish and pass away.

Now competent thinkers are agreed that there is no more permanent foundation for individual character and national greatness and worth than the exercise of a wise and just authority by the father. They base their judgment, not only on their own experience and the manifest fitness of things, but on the historical circumstances of persisting races and great nations. The late Frederick Denison Maurice, and many other writers, have expressed the firmest possible conviction that the greatness of ancient Rome had its origin in the family; that it began in reverence for fathers, and in faith in a guiding Providence. So long as this reverence and faith remained steadfast, and tended to become stronger, so long did Rome continue to increase in power and governing capacity.

A very different race from the Romans, the Jews, hold, and always have held the father of the family in peculiar veneration. "Honour thy father and thy mother that thy days may be long in the land," is as old as Moses, and as young as the youngest Hebrew child who has learnt to lisp the decalogue. It is due to this veneration for the father's authority, more, perhaps, than to any other single thing, that the Jews have preserved their peculiar identity unimpaired to the present day. If this be so, it is very clear that the authority of the father on the one hand, and the reverence of the child on the other, have opposed an almost miraculous resistance to the unparalleled forces of disintegration to which the Jewish race has been subjected for so many centuries.

Natural science in its widest significance confirms to the full the historical contentions of Mr. Maurice, the religious convictions of the Jew, and the philosophical and practical deductions of Mr. Herbert Spencer. One of the most powerful of social antiseptics and preservatives is paternal guidance and paternal authority. This principle, with an honest religious faith, makes a nation indestructible. The father is the thinker of the household; he is the moral reasoner; he is the representative of law rather than of sentiment, but still of law inspired by sentiment, clothed with sentiment, made worthy, not only of obedience, but of reverence and love, by sentiment. It is as plain as possible that nature intended the father to be the providence of the family—the true head. The tendency of some modern thought is to place the woman on a level of family authority with the man. This cannot be; it is contrary to nature. The woman is the complement of the man, but in no sense his equal in authority. Of course we are here stating the very broadest general principles; there is no space for details. But we are fully assured that Mr. Herbert Spencer's contention for the prime authority of the father is a contention of the very first importance from the point of view of the health and well-being of the family and of the health and stability of the State. Given a strong and wise central authority, together with a strong and wise paternal authority in every home, and you have one of the most essential of all the conditions of individual happiness as well as of State prosperity and State permanence. It is of the nature of things that in an old State, and with an increasing population, all kinds of family and social problems shall tend to become more complex, and more difficult of practical application to actual daily circumstances. But that being so, it is all the more essential to stand firmly upon, and constantly re-enunciate those eternal principles and rules which, like the rules of elementary arithmetic, are the persistent and unchanging bases of all the most complex developments of which social progress is capable.

